# Assays for Assessing *Mycobacterium avium* Immunity and Evaluating the Effects of Therapeutics

**DOI:** 10.3390/pathogens13100903

**Published:** 2024-10-15

**Authors:** Getahun Abate, Krystal A. Meza, Chase G. Colbert, Christopher S. Eickhoff

**Affiliations:** Division of Infectious Diseases, Allergy and Immunology, Department of Internal Medicine, School of Medicine, Saint Louis University, St. Louis, MO 63104, USAchristopher.eickhoff@health.slu.edu (C.S.E.)

**Keywords:** NTM, MAC, immunotherapy, T cells, macrophages, murine

## Abstract

In Europe and North America, the prevalence of pulmonary nontuberculous mycobacteria (NTM) is increasing. Most pulmonary NTM infections are caused by the *Mycobacterium avium* complex (MAC). Sadly, the treatment of pulmonary MAC is suboptimal with failure rates ranging from 37% to 58%. Therefore, there is a need to develop new therapeutics. Developing new immunotherapies and studying their interaction with standard or new drugs requires reliable assays. Four different assays including CFSE-based flow cytometry, in vitro protection assays, IFN-γ ELISPOT, and murine infection models were optimized using a reference strain of MAC (ATCC 700898) to help with the development of immunotherapies for MAC. Expansion of proliferating and IFN-γ producing human T cells is optimal after 7 days of stimulation with MAC at a multiplicity of infection (MOI) of 0.1, achieving a stimulation index of 26.5 ± 11.6 (mean ± SE). The in vitro protection assay for MAC works best by co-culturing T cells expanded for 7 days with MAC (MOI 1)-infected autologous macrophages. Aerosol MAC infection of mice allows measurement of the effects of the BCG vaccine and clarithromycin. IFN-γ ELISPOT assays with live MAC (MOI 3) stimulation of splenocytes from mice immunized with BCG help identify differences between unimmunized mice and mice immunized with BCG. In conclusion, multiple assays are available for use to identify MAC-specific effector T cells, which will help in the development of new therapeutics or vaccines against pulmonary MAC.

## 1. Introduction

Nontuberculous mycobacteria (NTM) are all mycobacteria except *M. tuberculosis* and *M. leprae*. Currently, over 190 species of nontuberculous mycobacteria (NTM) have been identified. There are more than 190 NTM species but only a few are clinically relevant [1]. NTM can affect any organ in the body, but in HIV-negative individuals, the pulmonary form is the most common form [2]. In countries where pulmonary tuberculosis (TB) is well controlled, the prevalence of pulmonary NTM in HIV-negative patients has been increasing over the last decade [2,3,4,5,6,7]. The reasons for the increasing prevalence of pulmonary NTM are not clearly known, but advances in extending the life expectancy of patients with underlying lung diseases such as cystic fibrosis and COPD, and the wide application of immunosuppressive medications in medicine, may play a role.

Based on data from 30 countries across six continents in 2008, *M. avium* complex (MAC) is the most common cause of pulmonary NTM, constituting 37% of isolates in Europe, 52% in North America, and 71% in Australia [8]. Regardless of the causative NTM species, the management of pulmonary NTM is extremely difficult. Treatment requires the use of multiple drugs for at least 12 months from the first culture-negative results, which, in most cases, means more than 18 months of treatment [9]. Despite treatment for several months, failure rates in the range of 30–40% have been reported [10,11,12]. Thus, there is an urgent need to develop new vaccines and therapeutics.

In the last decade, significant advances have been made in understanding TB immunology and bringing new vaccines and immunotherapeutics to clinical trials. The lessons learned in the TB field could be very useful for works on pulmonary NTM. Like TB, NTMs are intracellular bacteria, and their control relies primarily on mounting effective cell-mediated immunity [13,14]. Therefore, there is a need to have standardized assays that will help evaluate immunotherapies or vaccines against NTM. Because *M. avium* complex (MAC) is the most common cause of pulmonary NTM, we used a reference strain of MAC to optimize key immunological studies.

## 2. Materials and Methods

Four assays that help assess MAC-specific T cell responses and evaluate the anti-MAC activities of these T cells were optimized. These included (1) the flow cytometry assay, (2) IFN-γ ELISPOT, (3) the mycobacterial growth inhibition assay (also called in vitro protection assay), and (4) the murine MAC infection model.

### 2.1. Flow Cytometry

Connaught Bacillus Calmette Guerin (BCG), MAC (ATCC 700898), and MAC-whole lysate (WL) were used as antigens. The antibodies used included anti-γδ T cell receptor (TCR) antibody–phycoerythrin (PE) (clone 11F2), anti-γδ TCR APC (Clone B1), anti-αβ TCR antibody–fluorescein isothiocyanate (FITC) (clone B3), anti-CD3 antibody–peridinin chlorophyll protein (PerCP) (clone SK7), anti-CD4 Pacific Blue (clone RPA-T4), anti-CD8 antibody–PE-Cy7 (clone RPA-T8), and anti-IFN-γ APC antibody–Alexa Fluor 700 (clone B27). All antibodies were from BD Biosciences (Franklin Lakes, NJ, USA). Becton-Dickinson. Other reagents important for the assay included Carboxyfluorescein succinimidyl ester (CFSE; Molecular Probes, OR, USA), phorbol myristate acetate (PMA; Sigma-Aldrich, St. Louis, MO, USA), ionomycin (Sigma-Aldrich), and the Cytofix/Cytoperm kit (BD Biosciences). Human peripheral blood mononuclear cells (PBMCs) were obtained from healthy individuals who were purified protein derivative (PPD) skin test-positive or individuals with recent BCG vaccination through projects approved by the institutional review board.

We first optimized the concentrations of BCG, MAC, and MAC WL on samples from two volunteers. Optimized concentrations were used for the expansion of BCG-specific or MAC-specific T cells.

PBMCs were labeled for 15 min with 1 μmol/L CFSE (Molecular Probes) in PBS before being washed with RPMI 1640 medium, as recommended by the manufacturer. CFSE-labeled PBMCs (1 × 10^6^/mL) were stimulated with the different mycobacterial antigens mentioned above or were allowed to rest in a medium (1 mL of RPMI with 10% heat-inactivated pooled human AB serum [Sigma-Aldrich], L-glutamine [Fischer], and penicillin/streptomycin [Fischer Scientific,] Hampton, NH, USA) in polystyrene round-bottom tubes (17 × 100 mm; BD) for 7 days at 37 °C in 5% CO_2_. On day 7 of in vitro stimulation, the cells were restimulated with PMA (50 ng/mL) and ionomycin (750 ng/mL) in the presence of GolgiStop (0.7 μL/mL) for 2 h to maximize the intracellular expression of immune effector molecules already up-regulated by antigen-specific stimulation. Restimulated cells were washed with staining buffer and then stained to detect CD4, CD8, and γδTCR surface expression. These cells were subsequently permeabilized with Cytofix/Cytoperm solutions, as described by the manufacturer (BD), and then were stained to detect intracellular IFN-γ. Flow cytometric acquisition was performed on a BD SLR_II flow cytometer (BD Biosciences) instrument, and analyses were performed using FlowJo (Tree Star) software. A minimum of 10,000 events were acquired. The lymphocyte population was identified based on forward and side scatter. Then, CD3^+^ CD4^+^, CD3^+^ CD8^+^, and CD3^+^ γδ-TCR^+^ T cells were regated, and the CFSE low (CFSE^lo^) (proliferating) populations positive for IFN-γ were identified as effector subsets. The absolute numbers of effector populations were calculated by multiplying the percentage of each subset obtained with flow cytometry by the trypan blue-determined total viable cell counts.

### 2.2. In Vitro Protection Assay

BCG and MAC were used to infect macrophages. Other reagents used in the assay include saponin (Sigma, Tokyo, Japan), ADC enrichment (Difco, Detroit, MI, USA), and [^3^H]uridine (GE Healthcare incorporation, Chicago, IL, USA). PBMCs from PPD-positive or BCG-vaccinated healthy individuals were used.

Mycobacterial growth inhibition assays were performed as described previously [15]. First, we tested various mycobacterial concentrations for monocyte infection and the duration of the infection period before selecting a single MOI and duration of infection for further experiments. Briefly, adherent monocytes were infected overnight with MAC or BCG at an MOI of 3. Extracellular mycobacteria were removed by washing in warm RPMI medium 3 times. PBMCs expanded with BCG for 7 days were added to achieve an effector-to-target (E:T) ratio of 10:1, and co-cultures were incubated at 37 °C with 5% CO_2_ for 72 h. After 72 h, supernatants were aspirated, and target cells were lysed with 0.2% saponin to release intracellular mycobacteria. Saponin lysates were diluted 1:2 in 7H9 broth with 10% ADC enrichment medium and pulsed with 1 μCi of [^3^H] uridine per well. After incubation at 37 °C for 72 h, BCG or MAC incorporating the label was harvested onto glass fiber filter mats (Skatron, Sterling, Va.) and quantitated by liquid scintillation counting. Saponin lysates were also plated on 7H10 Middlebrook agar for colony-forming unit (CFU) determinations. The percentages of mycobacterial growth inhibition were determined using the following formula: % inhibition = 100 − [100 × (CFU or counts per minute (CPM) in the presence of BCG-stimulated T cells/CFU or CPM in the presence of medium-rested T cells)].

### 2.3. Murine Model

The animals used included 6–8-week-old BALB/c and C57BL/6 mice (The Jackson laboratories, Bar Harbor, ME, USA). BCG was used for vaccination, and MAC (ATCC 700898) was used for aerosol infection. Animal studies were approved by institutional animal care and use committee (IACUC) and performed in Association for Assessment and Accreditation of Laboratory Animal Care International (AALAAC) accredited facilities.

BCG vaccination: BCG administered intranasally (IN), subcutaneously (SC), or intradermally (ID) was used as a vaccine. Aliquots of BCG were thawed and pelleted by centrifuging at 3700 rpm for 15 min at 4 °C. The pellets were resuspended in phosphate buffer saline (PBS). Mice that received IN BCG had 1 × 10^7^ BCG delivered in 40 µL doses split between nostrils. Groups of mice that received SC BCG received 1 × 10^7^ bacteria in 100 µL into the base of the tail. Mice that received ID BCG had 1 × 10^7^ in 50 µL PBS delivered to the left side of shaved abdomens. DAR-901 was diluted to various doses (0.1 to 2.5 mg/dose) in PBS and administered in a total volume of 50 µL using insulin syringes (BD Biosciences, San Jose, CA, USA). Mice were anesthetized with a ketamine/xylazine cocktail intraperitoneally before IN or ID vaccination.

MAC challenge: Two to three weeks before mice experiments, aliquots of MAC (ATCC 700898) were thawed and cultured in fresh ADC-supplemented 7H9 media without Tween. On the day of challenge, mycobacterial suspensions were centrifuged, and the pellets were resuspended in PBS. The optical density (OD) of the suspension was measured at 600 nm. The suspension was diluted to adjust the optical density to an OD of 0.7. We estimated 3.1 × 10^7^ CFUs per OD unit based on results from previous titration experiments. MAC at an estimated final concentration of 2 × 10^7^ CFU/mL was added to the nebulizer and delivered via the aerosol route using the Glas-Col Inhalation Exposure System (IES) (Glas-Col Inc., Terre Haute, IN, USA). Some animals were euthanized immediately post-exposure to quantitate the delivery dose using the methods described above.

Quantifying lung CFUs after MAC infection: A total of 3–7 mice were sacrificed on day 0 and weeks 2, 4, 6, and 8 post-MAC infection. Lungs were homogenized in sterile PBS using a bead mill homogenizer, serially diluted, and plated in duplicate for CFU quantification on 7H11 agar media. For mice vaccinated with BCG, additional oleic–albumin–dextrose–catalase (OADC)-supplemented Middlebrook 7H10 agar media containing isoniazid at a concentration of 1 µg/mL was used to inhibit the growth of BCG. Plates cultured at 37 °C were read every week, and the CFU counts were finalized on week 4.

### 2.4. IFN-γ ELISPOT

BCG, live MAC (ATCC 700898), and MAC whole lysate were used as antigens. Cells resting in the medium were used as negative controls.

The assay was performed as we described previously using BD ELISPOT kits, according to the manufacturer’s recommendation [15]. Splenocytes (5 × 10^5^ cells/well) harvested from mice at different times were stimulated overnight with live BCG or MAC at a multiplicity of infection (MOI) of 3. After overnight incubation at 37 °C, ELISPOT plates were developed, and IFN-γ producing spots in each well were enumerated using a C.T.L. ImmunoSpot analyzer and software. The results are presented as spot-forming cells (SFCs, mean ± SE) per million splenocytes.

## 3. Results

**Figure 1:**
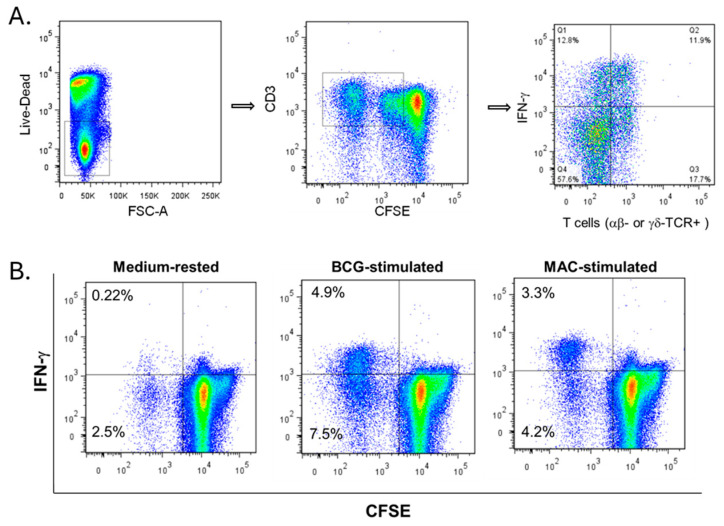
Typical FACS plot showing flow cytometric gating for proliferating and IFN-γ-producing T cells. (**A**) Gating strategy. (**B**) Proliferating and IFN-γ-producing T cells in PBMCs stimulated with BCG or M. avium compared to PBMCs rested in the medium. Similar results were obtained by gating SSC/FSC first followed by live-dead/CD3, CD3/γδ, CD4 or CD8, and then IFN-γ/CFSE.

**Figure 2:**
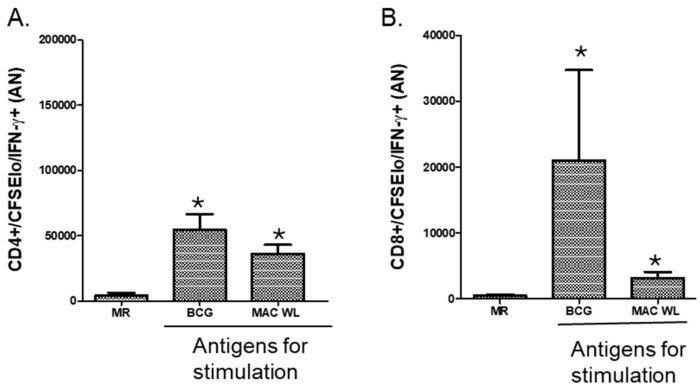
Stimulation of PPD-positive PBMCs with MAC leads to significant expansion of CD4 and CD8 T cells. PBMCs from PPD-positive volunteers (*n* = 5) were stimulated with BCG or MAC WL for 7 days. Medium-rested (MR) PBMCs were used as negative controls. There was a significant expansion of CD4 (**A**) and CD8 (**B**) T cells with both BCG and MAC WL (*, *p* < 0.05, Mann–Whitney U test).

**Figure 3:**
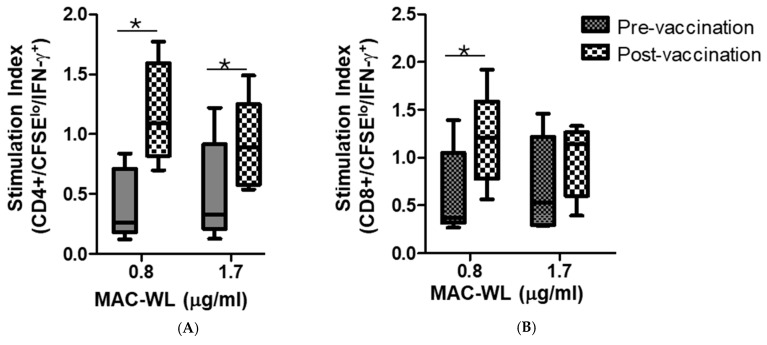
BCG vaccination in humans induces MAC cross-reactive T cells. Paired pre- and post-vaccination PBMCs from recently BCG-vaccinated volunteers living in the USA (*n* = 5) were used. PBMCs were labeled with CFSE and stimulated with different concentrations of MAC WL. Medium-rested PBMCs were used as negative controls. On day 7, cells were restimulated with PMA/ionomycin for 2 h, viable cells were counted, and cells were stained for surface and intracellular markers for the flow cytometry study. (**A**) Stimulation index of proliferating (CFSElo) and IFN-γ-producing CD4^+^ T cells. (**B**) Stimulation index of proliferating (CFSElo) and IFN-γ-producing CD4^+^ T cells. * *p* < 0.05 (Wilcoxon matched pairs test).

Measuring different effector functions of MAC-specific T cells may require the use of multiple assays. Figure 1 and Figure 2 show the applicability of flow cytometry to measure MAC immunity. Our gating strategy is shown in Figure 1. Proliferating and IFN-γ-producing effector T cells are seen in the left upper quadrant of the CFSE/IFN-γ plot. Figure 2 shows the cumulative results of MAC cross-reactive proliferating and IFN-γ-producing CD4 (Figure 2A) and CD8 (Figure 2B) T cells from PPD-positive individuals (*n* = 5) after 7 days of stimulation with live MAC-WL compared to BCG. These results show that there were significant expansions of CD4 (A) and CD8 (B) T cells with both BCG and MAC WL (*p* < 0.05, Mann–Whitney U test). PPD positivity could be from BCG vaccination or latent TB infection. Figure 3 shows similar flow cytometry results using pre- and post-BCG vaccination. Stimulation indices of MAC-specific proliferating and IFN-γ-producing CD4^+^ and CD8^+^ T cells were significantly higher in post-BCG-vaccination PBMCs (*p* < 0.05, Wilcoxon matched pairs test).

**Figure 4:**
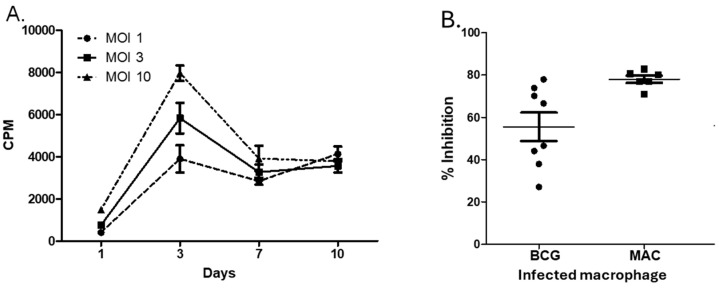
BCG-specific T cells cross-protect against MAC. Human monocytes from different volunteers were infected at different multiplicities of infection (MOI) overnight with MAC (ATCC 700898). Following infection, extracellular mycobacteria were washed away, and after various further incubation periods, macrophages were lysed and released mycobacteria by the ^3^H-uridine incorporation assay. MAC replicates inside macrophages, making them amenable to T cell effector functions. The results from a rapid ^3^H-uridine incorporation assay for MAC (**A**) were confirmed by CFU-plating of cultures at selected time points. (**B**) BCG-expanded T cells inhibit intracellular MAC potently as they inhibit intracellular BCG. PBMCs from BCG-vaccinated or latently individuals with TB infection were stimulated with the optimal concentration of live BCG in vitro for 7 days and co-cultured with autologous macrophages infected with either BCG (*n* = 8) or MAC. Residual mycobacteria were quantified 3 days after co-culture, and % inhibition was calculated by dividing the number of residual mycobacteria in the presence of BCG-stimulated PBMCs by the number of residual mycobacteria in co-cultures containing medium-rested PBMCs. BCG-expanded T cells inhibited intracellular MAV better than intracellular BCG (*p* < 0.01, Mann–Whitney U test).

To further assess the direct effector functions of MAC-specific T cells, we optimized and tested a mycobacterial growth inhibition assay that we previously used to measure effector functions of Mtb-specific T cells. For this assay to work, the mycobacteria have to grow inside normal human macrophages. Figure 4A shows that MAC grows inside human macrophages. Figure 4B shows that T cells expand with MAC in vitro and inhibit the growth of intracellular MAC, indicating the direct anti-MAC activities of BCG-expanded and MAC-expanded T cells.

**Figure 5:**
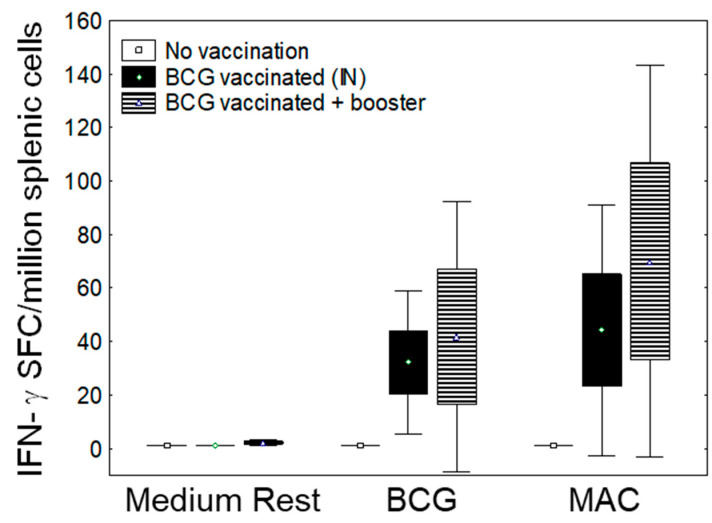
BCG vaccination renders MAC reactive immunity. Three groups of C57BL/6 mice were used. The first group (*n* = 3) was kept without vaccination. The second group was vaccinated with BCG (10 × 10^6^), intranasal (IN), and the third group was vaccinated with BCG, two doses 4 weeks apart. Mice were sacrificed 4 weeks after the last vaccination. Splenic cells were harvested and rested in the medium or stimulated overnight with live BCG and MAC, at a multiplicity of infection (MOI) of 3 in IFN-γ ELISPOT assays. Shown are mean ± SE from representative experiments expressed as IFN-γ spot-forming cells (SFCs) per million splenic cells. The number of IFN-γ SFCs following stimulation with BCG, MAC, and MAC-WL was significantly higher in BCG-vaccinated mice compared with unvaccinated mice (*p* < 0.05, Mann–Whitney U test). A second BCG vaccination did not significantly increase the number of mycobacteria-induced IFN-γ SFCs (*p* > 0.05).

**Figure 6:**
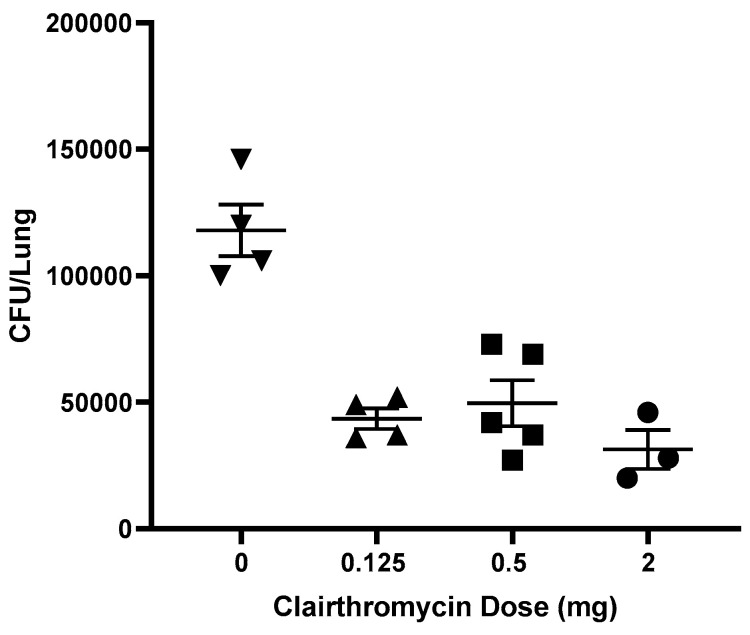
Effects of the anti-MAC drug on MAC growth in the lungs. Mice were infected with MAC, and two weeks after infection, clarithromycin at a concentration ranging from 0.125 mg to 2 mg per 20 g was started. Clarithromycin was administered 5 days a week via gavage between weeks 2 and 6 post-infection. All mice were euthanized six weeks after infection, their lungs were homogenized, and CFUs were quantified by culturing on 7H10 media. All doses of clarithromycin used decreased lung CFUs significantly (*p* < 0.05).

Animal models are important to evaluate MAC immunity induced or boosted by an intervention (e.g., vaccination or immunotherapy) and assess the effects of MAC immunity on MAC colony-forming units in the lungs following aerosol challenge. Figure 5 shows that mucosal BCG vaccination induces MAC immunity, as measured by IFN-γ ELISPOT using splenic cells stimulated with mycobacterial antigens overnight. The number of IFN-γ SFC following stimulation with BCG, MAC, and MAC-WL were significantly higher in BCG-vaccinated mice compared with unvaccinated mice (*p* < 0.05, Mann–Whitney U test). Figure 6 shows that 2 weeks of treatment with different doses of clarithromycin significantly inhibit the growth of MAC in the lungs, demonstrating the usefulness of a small animal model to assess therapeutics.

## 4. Discussion

Mycobacteria are primarily intracellular pathogens, and T cells are important for protection against mycobacterial infections [16]. The antimycobacterial activity of T cells is a balance between the potency of immune responses and the ability of mycobacteria to evade the immune system [17]. In patients with underlying lung diseases, it is believed that pulmonary MAC is associated with a lack of MAC-specific Th1 immune responses [18]. Our results show that multiple assays such as flow cytometry, IFN-γ ELISPOT, the mycobacterial-growth inhibition assay, and murine studies could be used in complementary fashion to identify interventions that induce or enhance MAC-specific immunity.

In healthy individuals, after the initial encounter with an antigen during immunization or natural infection, T cells pass through different phases, including programmed expansion, differentiation into effector cells, and programmed contraction [19,20]. Surprisingly, patients with pulmonary MAC have no change in CD8 memory T cells and lower levels of T effector memory cells compared with healthy controls [18]. Not all memory cells are useful to provide critical protective effector functions. Using *Listeria monocytogenes* infection in mice as a model, Lauvau et al. showed that both heat-killed and live *Listeria* species elicit memory CD8^+^ T cells, but only those induced by live *Listeria* species proliferate and provide protective effector functions [21]. Similarly, a study of individuals with HIV infection indicated that long-term non-progressors had markedly higher levels of effector T cells capable of expansion and effector molecule expression, compared with individuals with HIV infection and AIDS [22]. Therefore, reliable measurements of pathogen-specific T cell responses should identify subsets that both proliferate and produce effector molecules when stimulated with a relevant antigen. Our results showed that a CFSE-based flow cytometry assay can reliably measure the number of proliferating subsets of T cells expressing effector molecules relevant to protect against MAC.

Based on the percent of IFN-γ or IL-4 CD4^+^ T cells, patients with pulmonary MAC appear to have mixed Th1 and Th2 T cell responses when peripheral T cells are stimulated with MAC in vitro [23]. These mixed responses may not be optimal responses to control mycobacterial infection. Our results, using assays on human samples and murine studies, show that vaccines such as BCG can induce a Th1 immune response with increased absolute numbers of proliferating and IFN-γ-producing T cells, indicating the potential to use BCG as a protective vaccine in high-risk individuals or for immunotherapy of pulmonary MAC patients [15].

BCG intradermal challenge and quantification of the number of bacteria at the challenge site have been proposed as a way to evaluate the direct effector functions of new TB-vaccine-induced immunity [24,25]. There are no similar human challenge models for the study of pulmonary MAC, and the use of macrophage cell lines such as THP-1 cells does not allow for co-culturing with effector T cells. Therefore, we used mycobacterial growth inhibition assay, which we used before to evaluate new TB vaccines [26,27,28,29]. In the mycobacterial growth inhibition assay, T cells stimulated in vitro are co-cultured with MAC-infected autologous macrophages, and in 3 days, residual intracellular mycobacteria are quantified. It is expected that effector T cells inhibit the growth of intracellular mycobacteria; therefore, the number of residual MAC will be lower in co-cultures that include effector T cells compared with controls. Our results showed that this assay could measure effector functions of T cells induced by BCG vaccination.

Animal models are essential to advance studies on MAC immunity, develop effective vaccines, and test new therapeutics. Different mouse strains including BALB/c, C57BL/6, Beige, nude, and C3HeB/FeJ have been used to test vaccines or drugs against pulmonary MAC [30,31]. It appears that all these strains of mice may be used to test vaccines. However, BALB/c mice are preferable for studies on anti-MAC drugs [30]. We evaluated MAC immunity and infection in both BALB/c mice. The effects of drug treatment after aerosol infection of mice could be easily evaluated by quantifying lung CFUs after treatment. The same model could be used for evaluation of immunotherapies.

## 5. Conclusions

The prevalence of pulmonary NTM is increasing, and MAC is the most common cause of pulmonary NTM. Treatment for pulmonary MAC is long, and failure rates are high. There is a need to develop vaccines and host-directed therapies. The development of new vaccines or therapeutics requires reliable immunological assays. One assay may not be enough to evaluate the different aspects of MAC immune responses. Assays to measure MAC-specific T cells and their direct effector functions on intracellular mycobacteria or their ability to limit growth in the lungs provide complimentary results, which will enable the evaluation and selection of new vaccines and therapeutics. Our assays for human and murine studies will help evaluate not just MAC T cell responses but the direct effector functions of these T cells.

## Data Availability

Data may be obtained, upon request, from the corresponding author.
